# Silence of the lungs: comparing measures of slow and noncommunicating lung units from pulmonary function tests with computed tomography

**DOI:** 10.1152/japplphysiol.00340.2024

**Published:** 2024-08-08

**Authors:** Christopher Short, Thomas Semple, Mary Abkir, Simon Padley, Mark Rosenthal, Paul McNally, Harm Tiddens, Daan Caudri, Andrew Bush, Jane C. Davies

**Affiliations:** ^1^National Heart and Lung Institute, Imperial College London, London, United Kingdom; ^2^Royal Brompton and Harefield Hospitals, Guys and St Thomas’ Trust, London, United Kingdom; ^3^European Cystic Fibrosis Society, Lung Clearance Index Core Facility, London, United Kingdom; ^4^Paediatrics and Child Health, Imperial College London, London, United Kingdom; ^5^RCSI University of Medicine and Health Sciences, Dublin, Ireland; ^6^Children’s Health Ireland, Dublin, Ireland; ^7^Department of Paediatric Pulmonology and Allergology, Erasmus MC—Sophia Children’s Hospital, Rotterdam, The Netherlands; ^8^Department of Radiology and Nuclear Medicine, Erasmus MC, Rotterdam, The Netherlands; ^9^Thirona, Nijmegen, The Netherlands

**Keywords:** air trapping, collateral ventilation, cystic fibrosis (CF), pulmonary function testing (PFT), pulmonary physiology

## Abstract

Multiple breath washout (MBW) has successfully assessed the silent lung zone particularly in cystic fibrosis lung disease, however, it is limited to the communicating lung only. There are a number of different pulmonary function methods that can assess what is commonly referred to as trapped air, with varying approaches and sensitivity. Twenty-five people with cystic fibrosis (pwCF) underwent MBW, spirometry, body plethysmography, and spirometry-controlled computed tomography (spiro-CT) on the same day. PwCF also performed extensions to MBW that evaluate air trapping, including our novel extension (MBW_ShX_), which reveals the extent of underventilated lung units (UVLU). In addition, we used two previously established 5-breath methods that provide a volume of trapped gas (VTG). We used trapped air % from spiro-CT as the gold standard for comparison. UVLU derived from MBW_ShX_ showed the best agreement with trapped air %, both in terms of correlation (*R*_S_ 0.89, *P* < 0.0001) and sensitivity (79%). Bland–Altman analysis demonstrated a significant underestimation of the VTG by both 5-breath methods (−249 mL [95% CI −10,796; 580 mL] and −203 mL [95% CI −997; 591 mL], respectively). Parameters from both spirometry and body plethysmography were suboptimal at assessing this pathophysiology. The parameters from MBW_ShX_ demonstrated the best relationship with spiro-CT and had the best sensitivity compared with the other pulmonary function methods assessed in this study. MBW_ShX_ shows promise to assess and monitor this critical pathophysiological feature, which has been shown to be a driver of lung disease progression in pwCF.

**NEW & NOTEWORTHY** We consider the term “trapped air” either in the use of imaging or pulmonary function testing, something of a misnomer that can lead to an inaccurate assessment of an important physiological feature. Instead, we propose the term underventilated lung units (UVLU). Of the many pulmonary function methods we used in this study, we found that the use of multiple breath washout with short extension (MBW_ShX_) to be the best nonimaging method.

## Graphical abstract

**Figure:**
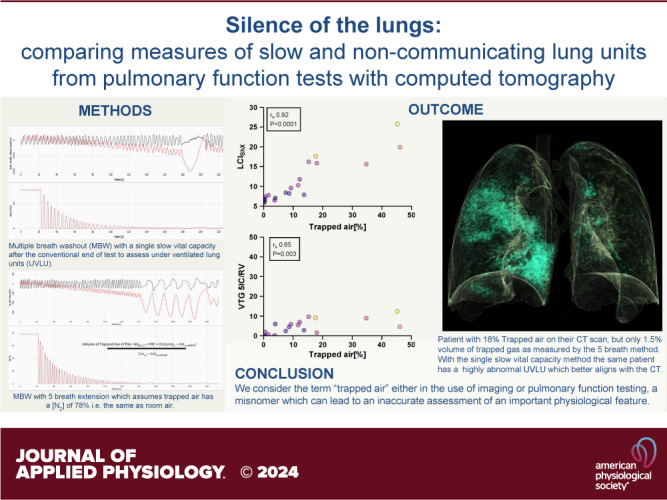

## INTRODUCTION

Accurately assessing peripheral airways (<2 mm in diameter) using pulmonary function tests has been a challenge for decades leading to the term “silent zone.” Forced expiratory volume in 1 s (FEV_1_) obtained from the commonest pulmonary function test, spirometry, is mainly derived from the proximal airways, with as little as 10% reflecting the peripheral airways (1, 2). Markers of peripheral airway function from spirometry such as FEF_75_ or FEF_25–75_ are more useful to help assess the silent lung zone, but their inherent variability renders them somewhat unreliable. Body plethysmography uses several metrics in an attempt to shine a light on the silent lung zone, but lacks the required sensitivity to be clinically useful (3).

Due to this and other factors, multiple breath washout (MBW) has had a resurgence in the past couple of decades, being successfully deployed as a primary and secondary outcome in cystic fibrosis (CF) clinical trials (4–6). More recently, MBW has been implemented clinically with some specialist centers using it in routine clinical practice. MBW is a sensitive measure of ventilation efficiency, which is an umbrella term for ventilation inhomogeneity, physiological deadspace, and gas mixing efficiency. The primary parameter is the lung clearance index (LCI), which is a derived index of the number of lung volume turnovers required to clear the chosen tracer gas to a prespecified concentration, conventionally 2.5% of its starting concentration (hence often termed LCI_2.5_). Although LCI_2.5_ is far more sensitive in assessing the silent lung zone, it is only a measure of the communicating lung, as signal from occluded or collapsed lung units will not be captured. This limitation had previously been addressed, at least partially, whereby after the conventional end of MBW the subject performed five vital capacity (maximal) breaths, from which the exhalate was collected in a gas sampling bag; the total volume of N_2_ collected was termed the volume of trapped gas (VTG). The volume of trapped gas has been shown to be more sensitive to predict early bronchospasm after allergy provocation in subjects with asthma compared with LCI_2.5_ or spirometry (7). The volume of trapped gas also had better sensitivity to detect lung disease in children with CF compared with either LCI_2.5_ or spirometry (8).

Modern computing has allowed breath by breath tracking of expired gases to allow automatic plotting of washout curves in real time. This led to the discarding of gas sampling bags and consequently volume of trapped gas could not be calculated. Subsequently, a calculation to assess volume of trapped gas was developed without the need of a gas sampling bags (9). It was shown to be feasible and repeatable in children and sedated infants (10) and even demonstrated a degree of gas trapping in healthy individuals (11). However, a fundamental assumption of this method is that the gas mobilized by the five inspiratory or vital capacity breaths has an [N_2_] of ∼78%. However, if these lung units are partially ventilated, i.e., through collateral channels, volume of trapped gas will be underestimated. For example, Gustafsson (11) postulated that “If, however, the true mean N_2_ concentration of the noncommunicating lung volumes were only ∼40%, due, e.g., to collateral ventilation, then the true volume of trapped gas, N_2_ result would be twice as high as the currently.”

Recently, we developed an adaptation to MBW that we termed the Short extension (MBW_ShX_). This utilizes a single slow vital capacity after the conventional end of MBW to provide a functional measure of the extent of underventilated lung units (UVLU) (12). UVLU can be used as a standalone marker or when combined with LCI_2.5_ it can provide a global measure of pulmonary function termed (LCI_ShX_). MBW_ShX_ requires no assumptions of N_2_ concentration and does not try to calculate the volume of trapped gas. Instead, it uses the change in the [N_2_] brought on by the slow vital capacity, to provide a functional representation. Pilot data of the method showed that UVLU could not be predicted by the baseline LCI_2.5_ and thus provides additional information by way of signal from distal airway disease. LCI_ShX_ had a similar intertest repeatability to LCI_2.5_ but a larger change around periods of CF pulmonary exacerbation. LCI_ShX_ also demonstrated a good relationship with computed tomography (CT) disease scores and significant agreement with the extent of mucus plugging, unlike LCI_2.5_. However, to date, MBW_ShX_ and the previous methods of assessing the volume of trapped gas have not been directly compared and have not been evaluated against an independent sensitive marker of air trapping/UVLU.

## AIMS AND HYPOTHESIS

We hypothesize that the novel parameters derived from MBW_ShX_ are more sensitive to detect air trapping/UVLU and correlate significantly better with CT air trapping than routine pulmonary function parameters. We aimed to test this hypothesis opportunistically using a CF cohort who were recruited to an ongoing phase IV clinical trial. Second, we aimed to use these data to challenge the assumption that the [N_2_] within the “trapped air” spaces is 78% and the same within all “trapped air spaces.”

## METHODS

### Participants

We opportunistically studied 25 people with cystic fibrosis (pwCF) who were recruited as part of an ongoing phase IV clinical trial (RECOVER clinicaltrials.gov-NCT04602468) where spirometry, MBW, and spirometry-controlled CT (spiro-CT) were performed. In addition, participants performed body plethysmography to calculate lung volumes. Ethical approval was granted by Health and Care Research Wales Ethics Committee (21/LO/0224). Informed written consent and age-appropriate assent was obtained from each parent, guardian, and/or participant.

### Spirometry

Spirometry was performed by participants on the same day as spiro-CT and MBW, using either a Nuvoair or Spirobank spirometer as per their usual home spirometry measures. Flow loops were analyzed by at least one clinical respiratory physiologist according to ERS/ATS standards (13). Percent predicted values were calculated using the global lung initiative 2012 reference ranges (14). Percent predicted was used for all spirometry parameters. Parameters that were used are forced expiratory volume in 1 s (FEV_1_), forced vital capacity (FVC), forced expiratory flow at 25%–75% of the FVC (FEF_25–75%_), and flow when 75% of FVC has been exhaled (FEF_75%_).

### Body Plethysmography

Body plethysmography was performed using a Vyaire system following clinical protocols within our institution. Three acceptable maneuvers were performed, with the best maneuver performed being used as the final result. Test were performed by a respiratory physiologist according to ERS/ATS standards and percent predicted values and *Z*-scores were calculated using the global lung initiative 2021 reference ranges (15). Parameters used to define “trapped air” were the residual volume/total lung capacity shown as a percentage. We also used the parameter functional residual capacity (FRC) from body plethysmography-FRC from MBW. Since the FRC from MBW represents the communicating lung and the FRC from body plethysmography represents all air inside the thoracic cavity, the difference is hypothesized to represent “trapped air.”

### Multiple Breath Nitrogen Washout

Multiple breath nitrogen washout (MBWN_2_) was performed using the ExhalyzerD (Ecomedics AG, Switzerland) with MBW results calculated using the cross-sensor error corrected software (Spiroware v.3.3.1.). Post hoc quality control was performed in accordance with the ERS/ATS consensus statement and central over-reading standards (16, 17). LCI_2.5_ was the only parameter used from conventional MBWN_2_.

### Extensions to MBW

MBW extensions (MBW_ShX_ and 5 inspiratory capacity and 5 expiratory vital capacity breaths) were performed in a random order using a random number generator after the conventional end of MBW run had been reached (with at least 3 tidal breaths under <2.5% of the starting N_2_ concentration). MBW_ShX_ was performed and quantified as described previously (12), both UVLU and LCI_ShX_ are the parameters used. Two 5 inspiratory methods were used: *1*) 5 Inspiratory capacity breaths (5IC): 4 inspiratory capacity breaths, followed by a single slow vital capacity breath, and *2*) 5 vital capacity (5VC): 5 slow vital capacities. The 5IC and 5VC data were analyzed using a derivative of the Gustafsson method, see the supplementary material of the ERS/ATS consensus statement (16). Volume of trapped gas can be plotted over any lung volume and converted into a % (e.g., FRC or VC), and in this study the residual volume (RV) acquired from the Spiro-CT was used as the denominator for direct comparison. Parameters used are VTG/RV% and VTG/VC for both 5-breath methods.

### Spirometry-Controlled Computed Tomography Scans

Spiro-CT images were acquired at both total lung capacity (TLC) and at residual volume while using real-time spirometric monitoring computer software. When the required inspiratory and expiratory target threshold volumes were reached, image acquisition was initiated by the physiologist signaling to the CT technician. Both the radiology and physiology teams performing the study were trained and certified by the Erasmus European CF society CT core facility. All scans were performed on a dual source CT scanner (Siemens Definition FLASH) using a low dose, CF-specific protocol. Inspiratory and expiratory spiro-CT images were scored using the validated PRAGMA-CF scoring system (18). Individual disease component scores and an airway disease composite score are calculated from the images acquired at TLC. Trapped air percentage (trapped air %) is calculated from the image acquired at residual volume. Further information can be found in the Supplemental Material (see https://doi.org/10.6084/m9.figshare.26362468).

### Assessing Discriminatory Power of Physiological Measures for Air-Trapping Compared with to spiro-CT

Sensitivity, specificity, positive and negative predictive values, and accuracy (19) of pulmonary function parameters to detect trapped air were calculated using spiro-CT as the “gold standard.” For this analysis, volume of trapped gas was plotted over vital capacity (VTG/VC%) as currently, there are no reference ranges to determine discriminatory power when plotted over RV.

*Calculations*

Sensitivity = True Positive/(True Positive + False Negative)

Specificity= True Negative/(False Positive + True Negative)

Accuracy=(True Positive+True Negative)/(True Positive+True Negative+False Positive+False Negative)

Positive Predicted Value=(Sensitivity×Prevalence)/ [(Sensitivity × Prevalence)+((1−Specificity)×(1−Prevalence))]

Negative Predicted value=(Specificity×(1−Prevalence))/[((1−Sensitivity)×Prevalence)+(Specificity×(1−Prevalence))]

### Statistical Analysis

This sample was opportunistically obtained as part of an ongoing phase IV clinical trial. Prism (v.9.1, GraphPad Software, Inc, San Diego, CA) was used for statistical analysis. Normality testing was performed using the Shapiro–Wilk test. Wilcoxon matched-pairs signed rank test was used to assess the added time needed for the various MBW extensions. Spearman’s rank order test was used to assess correlations between MBW outcomes and imaging outcomes, further linear regression analysis was performed for selected parameters. We compared the strength of Spearman’s correlation coefficients using Fisher’s *Z*-test on z-transformed correlation coefficients (MedCalc Software Ltd., Ostend, Belgium). χ^2^ analysis was performed to compare the sensitivity of UVLU against other pulmonary function parameters to detect air trapping. To calculate the modeled N_2_ concentration in the “trapped air” spaces, we used a molarity calculator (Graph Pad Software, Inc., San Diego, CA); description and example are provided in the Supplemental Material (https://doi.org/10.6084/m9.figshare.26362468). Data are presented as median (range).

## RESULTS

### Baseline Characteristics and Feasibility

Baseline characteristics of the study cohort are summarized in Table 1. All 25 subjects successfully performed MBW, body plethysmography, spirometry, and spiro-CT. Subjects performed MBW extensions with success rates of 100% for MBW_ShX_, 72% (18/25) for the 5IC, and 80% (20/25) for 5VC breaths. The duration of an MBW run was 123 s (range 72–512 s) and was extended by a median of 24 s (13–40 s, *P* < 0.0001) with MBW_ShX_, 73 s (40–96 s, *P* < 0.0001) with the 5IC, and 90 s (68–153 s, *P* < 0.0001) with 5VC breaths. Unsurprisingly, both the 5 breath methods were significantly longer than MBW_ShX_ (*P* < 0.0001 respectively).

**Table 1.** Baseline characteristics of people with cystic fibrosis recruited in this study

Baseline Characteristics	
*n*	25
Male %	13 (52%)
Age	13 (7–58)
Spirometry	
ppFEV_1_	94% (61%–123%)
ppFVC	101% (74%–128%)
ppFEF_25-75_	74% (30%–120%)
ppFEF_75_	75% (23%–141%)
MBW_ShX_	
LCI_2.5_	6.9 (5.8–22.8)
LCI_ShX_	8.1 (5.9–30.5)
UVLU	0.8 (0.1–8.6)
Volume of trapped gas (VTG)	
5IC VTG/RV	2.8% (0.1%–12.4%)
5VC VTG/RV	4.4% (0.1%–14.9%)
Body plethysmography	
RV/TLC%	25.8% (19.0%–43.4%)
FRC_Pleth_-FRC_MBW_/RV %	43.95% (1.3%–104%)
Spiro-CT	
Airway disease score %	2.3% (0.3%–12.2%)
Bronchiectasis %	1.7% (0%–9.4%)
Mucus plugging %	0% (0%–2.7%)
Trapped air %	14.9% (0%–46.1%)

5IC, 5 inspiratory capacity breaths; 5VC, 5 vital capacity; FRC, functional residual capacity; LCI, lung clearance index; MBW_ShX_, Short extension multiple breath washout; ppFEF_25–75%_, percent predicted forced expiratory flow at 25–75% of the forced vital capacity (FVC); FEF_75%_, percent predicted forced expiratory flow when 75% of FVC has been exhaled; ppFEV_1_, percent predicted forced expiratory volume in 1 second; ppFVC, percent predicted forced vital capacity; RV, residual volume; Spiro-CT, spirometry-controlled computed tomography; TLC, total lung capacity; UVLU, under-ventilated lung units; VTG, volume of trapped gas.

### Agreement of Pulmonary Function Parameters with Trapped Air % from Spiro-CT

**Figure 1.**
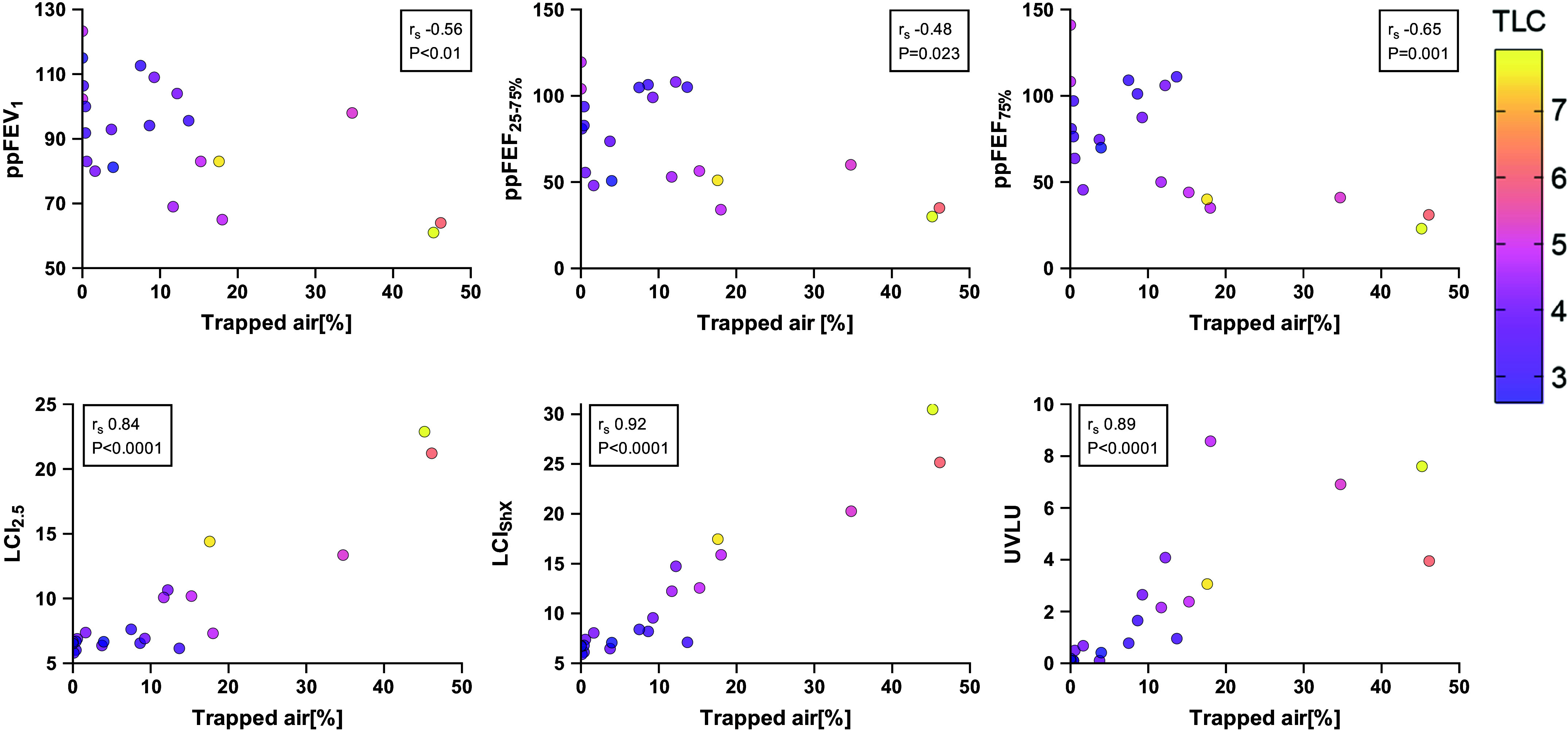
Correlations between trapped air % from spirometry-controlled computed tomography and pulmonary function parameters including percent predicted (pp) forced expiratory volume in 1 second (FEV_1_), forced expiratory flow at 25–75% of the forced vital capacity (FVC) (FEF_25–75%_), flow when 75% of FVC has been exhaled (FEF_75%_) from spirometry and derivatives of Short extension multiple breath washout (MBW_ShX_). Spearman’s correlation coefficient was used. Individual dots are color coded for a patient’s total lung capacity (TLC) which is measured in liters and is consistent on all scatter plots. UVLU, underventilated lung units.

**Figure 2.**
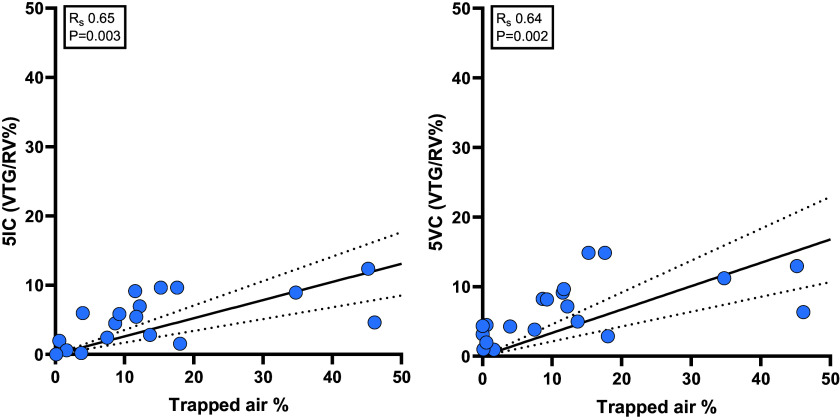
Spearman’s correlation coefficient of the volume of trapped gas (VTG) over residual volume % from the 5 inspiratory capacity breaths (5IC) method (*left*) and 5 vital capacity (5VC) method (*right*) with trapped air % from spirometry-controlled computed tomography. There is a clear under-read of the of VTG/residual volume (RV)% from both 5-breath methods compared with the trapped air %. Straight black line is a linear regression line, with dashed lines either side the 95% confidence intervals.

We used trapped air % from spiro-CT as the standard against which to assess our physiological outcomes. All MBW_ShX_ parameters demonstrated a significant correlation (LCI_2.5_ *R*_s_ 0.84, *P* < 0.0001; LCI_ShX_ *R*_s_ 0.92, *P* < 0.0001; UVLU *R*_s_ 0.89, *P* < 0.0001) (Fig. 1). Significant, although weaker, correlations were also seen with the VTG/RV% from both the 5IC and 5VC method (*R*_s_ 0.65, *P* = 0.003 and *R*_s_ 0.64, *P* = 0.002 respectively) (Fig. 2). There was significant correlations with ppFEV_1_ (*R*_s_ −0.56, *P* = 0.005) (Fig. 1), ppFEF_75_ (*R*_s_ −0.65, *P* = 0.001), ppFEF_25–75_ (*R*_s_ −0.48, *P* = 0.023) but not ppFVC (*R*_s_ −0.37, *P* = 0.075). Neither the FRC comparison method (*R*_s_ −0.24, *P* = 26) or the RV/TLC% (*R*_s_ 0.30, *P* = 0.16) had a significant agreement with trapped air %. Next, we compared the correlation coefficients of these parameters and found that UVLU had a significantly stronger correlation than VTG/RV% from either 5-breath method (*P* = 0.049 and *P* = 0.038, respectively). UVLU also demonstrated a significantly stronger correlation than the FRC comparison method (*P* < 0.0001), RV/TLC Ratio (*P* < 0.001), ppFEV_1_ (*P* < 0.009), and ppFEF_25–75_ (*P* < 0.0001).

### Discriminatory Power to Detect Trapped Air on Spiro-CT

The presence (>0%) of trapped air on spiro-CT was 19/25 giving a prevalence of 76%. The sensitivity, specificity, accuracy, and positive/negative predictive values of physiological parameters for air trapping on spiro-CT was calculated (Table 2). UVLU performed well across the board for all these parameters. Next, we directly compared the sensitivity of UVLU to the sensitivity of the other pulmonary function parameters. We found that UVLU had significantly greater sensitivity than LCI_2.5_ (*P* = 0.02), VTG/VC% from both 5 breath methods (*P* = 0.029 and *P* = 0.043 respectively), the FRC comparison method (*P* < 0.0001), RV/TLC ratio (*P* < 0.0001), FEV_1_ (*P* = 0.0003), and FEF_25–75_ (*P* < 0.022).

**Table 2.** Sensitivity, specificity, positive and negative predictive value as well as accuracy of nonimaging parameters to predict trapped air, with the trapped air % from spirometry-controlled computed tomography used as the gold standard and comparitor for this analysis

Parameter	Sensitivity, %	Specificity, %	Positive Predictive Value, %	Negative Predictive Value, %	Accuracy, %
ppFEV_1_	28	83	84	27	42
ppFVC	11	83	68	23	29
ppFEF_25–75%_	63	75	90	37	67
ppFEF_75%_	61	50	81	26	58
RV/TLC%	11	100	100	26	32
LCI_2.5_	47	100	100	38	60
LCI_ShX_	63	100	100	46	72
UVLU	79	100	100	60	80
5IC VTG/VC	47	100	100	25	55
5VC VTG/VC	50	100	100	37	61

5IC, 5 inspiratory capacity breaths; 5VC, 5 vital capacity; LCI, lung clearance index; ppFEF_25–75%_, percent predicted forced expiratory flow at 25–75% of the forced vital capacity (FVC); FEF_75%_, percent predicted forced expiratory flow when 75% of FVC has been exhaled; ppFEV_1_, percent predicted forced expiratory volume in 1 second; ppFVC, percent predicted forced vital capacity; RV, residual volume; TLC, total lung capacity; UVLU, under-ventilated lung units; VTG, volume of trapped gas.

### Comparing Volumes of Trapped Gas from MBW and Spiro-CT to Investigate the Validity of Assuming Exhaled N_2_ Concentrations of 78%

Although there were significant agreements between trapped air % and VTG/RV% for both 5-breath methods, there was a large discrepancy between the calculated volumes as shown in Fig. 2. Bland–Altman analysis of the volume of trapped gas from the 5IC method compared with trapped air (mL) showed a bias of −249 mL with wide 95% limits of agreement of (−1,079 to 580 mL, Supplemental Fig. S3). Similar under-reading results were observed in the 5VC method with a bias of −203 mL and 95% limits of agreement (−997 to 591 mL, Supplemental Fig. S3).

**Figure 3.**
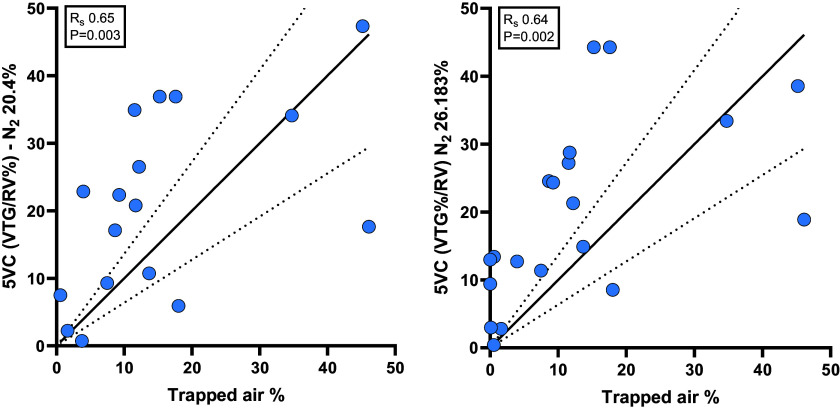
We used the linear regression slope values from Fig. 2, to update the fundamental assumption of 78% N_2_ (16) concentration to find a better agreement between the 5-breath methods and trapped air %. For the 5 inspiratory capacity breaths (5IC) method, the 1/slope value was 3.823 whereas for the 5 vital capacity (5VC) method, the 1/slope value was 2.979. Therefore, we recalculated the volume of trapped gas (VTG)/residual volume (RV)% for the 5IC method using a N_2_ of 20.40% (78/3.823) and 5VC breaths using a N_2_ of 26.183% (78/2.979) (Fig. 4). As would be expected, the correlation coefficient for both parameters stayed the same, but now the agreement on the extent of trapped air % was almost perfect with a 1/slope 1.00 for both parameters.

**Figure 4.**
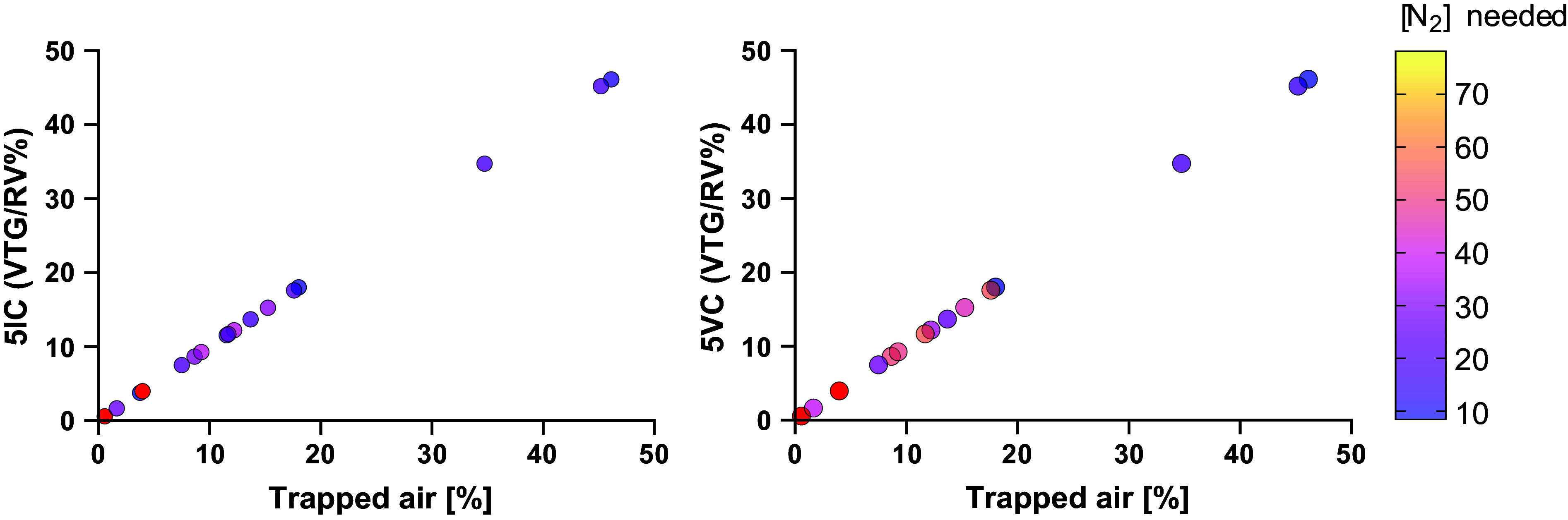
As seen in Fig. 3, a fixed correction factor applied to the assumption of N_2_ concentration of “trapped air” spaces still did not improve the correlation coefficient or remove large outliers, when comparing volume of trapped gas with trapped air%. Therefore, we used a molarity calculator to model the N_2_% for an individual patient that was likely present in the “trapped air” spaces which would generate a perfect agreement with trapped air % from spirometry-controlled computed tomography. Each dot is color coded for the N_2_% modeled to generate that perfect agreement. Note the patients in red had a larger volume of trapped gas (VTG) over residual volume % (RV%) than trapped air %, therefore N_2_ concentration could not be calculated. The range for N_2_% for the 5 inspiratory capacity breaths (5IC) method was 3.5% to 34.8% and for the 5 vital capacity (5VC) was 8.6% to 55.3%. Without having the accompanying trapped air% values calculating an accurate VTG/RV% is not possible.

To explore the reasons for this discrepancy, we used linear regression analysis constrained through zero to calculate the slope (Fig. 3). For the 5IC method, the 1/slope value was 3.823 whereas for the 5VC method the 1/slope value was 2.979. Therefore, we recalculated the VTG/RV% for the 5IC method using a N_2_ of 20.40% (78/3.823) and 5VC breaths using a N_2_ of 26.183% (78/2.979) (Fig. 4). As would be expected, the correlation coefficient for both parameters stayed the same (*R*_s_ 0.65, *P* = 0.003 and *R*_s_ 0.64, *P* = 0.002 respectively), but now the agreement on the extent of trapped air % was almost perfect with a 1/slope 1.00 for both parameters.

Next, we went one step further, using the trapped air % from spiro-CT and worked backward to calculate the N_2_ concentrations for which each maneuver would achieve a perfect agreement. Using a molarity calculator, the range of N_2_% for the 5IC method was 3.5% to 34.8% and for the 5VC was 8.6% to 55.3% (Fig. 4). For three patients, an accurate [N_2_] within the “trapped spaces” could not be calculated for either 5IC or 5VC breaths as the original volume of trapped gas (mL) was larger than the trapped air (mL) from spiro-CT.

## DISCUSSION

In this study, we assessed pulmonary function parameters from standard spirometry, MBW, and body plethysmography for their ability to detect and quantify an important pathophysiological feature (20) of CF lung disease, often referred to as “trapped air.” We found that parameters from MBW_ShX_ had the best agreement with trapped air% from spiro-CT, and that two previously published 5-breath methods both correlated less well with, and under-reported, the volume of trapped gas. We demonstrate this is likely a consequence of a flaw in the assumption that what is mobilized by either 5-breath method has a [N_2_] of ∼78%. We demonstrate that modeled N_2_ concentration was on average much lower within these “trapped” spaces and was highly variable between patients. Of course, there will be variance across the lung and indeed between different “trapped air spaces,” but here we present an average of those spaces for each individual patient.

### Interpretation of Results

All methods had a very high-performance success rate, all but 2 with 100%. The lowest success rates were with the 5IC and 5VC methods with 72% and 80% respectively, and this was because of unsuccessful attempts with younger participants aged 7–11 yr old. The parameters from body plethysmography (FRC comparison and RV/TLC%) were quickly identified as unreliable, as they were the only parameters that did not demonstrate significant correlations with the trapped air % from spiro-CT. They also possessed poor sensitivity and accuracy, supporting the contention from Rosenthal (3) that body plethysmography did not hold short- or long-term utility for monitoring CF lung disease. Spirometric parameters of small airways disease (FEF_75_ and FEF_25–75_) performed reasonably well considering the physiological limitations of the technique and the fact that they do not reflect “trapped air.” They showed moderate correlations with trapped air % and had a good sensitivity, but the specificity of these parameters is questionable, as they were the only parameters to identify false positives. The volume of trapped gas from either the 5IC or 5VC methods demonstrated only a moderate correlation with spiro-CT and had a poor sensitivity and accuracy when detecting “trapped air” (Table 2). We attribute the under-reporting to the fundamental assumption that what is mobilized by either 5-breath method has a N_2_% of ∼78%. When we tested this assumption by applying a standardized correction factor, we reduced the systemic large under-reading bias, but it did not resolve the large outliers seen in our sample. This fixed correction also caused a slight over-reading bias in subjects with mild to moderate air trapping (∼10%), because of constraining the linear regression through zero.

We further hypothesized that this was due to individual variations in the [N_2_] of the trapped air spaces due to processes such as collateral ventilation and lung units having slower time constants for ventilation than standard tidal breathing (21). We modeled the N_2_ concentration needed in these “trapped air” spaces to generate a perfect agreement with trapped air % from spiro-CT and confirmed a wide range of N_2_ concentrations. We conclude that any test requiring on an assumed, fixed [N_2_] will not generate accurate measurements. There is of course also variation between “trapped air” spaces in the same individual, which further highlights the challenges of using any such assumption. The parameters from MBW_ShX_ (LCI_ShX_ and UVLU) do not require any assumptions of [N_2_] as they use only the observed signal in the [N_2_] generated by the slow vital capacity. They were feasible in 100% of participants and added only ∼30 s to an MBW trial. They performed well, with the strongest correlations with trapped air % and the greatest sensitivity and accuracy of any parameters in this study.

### Theoretical Considerations

**Figure 5.**
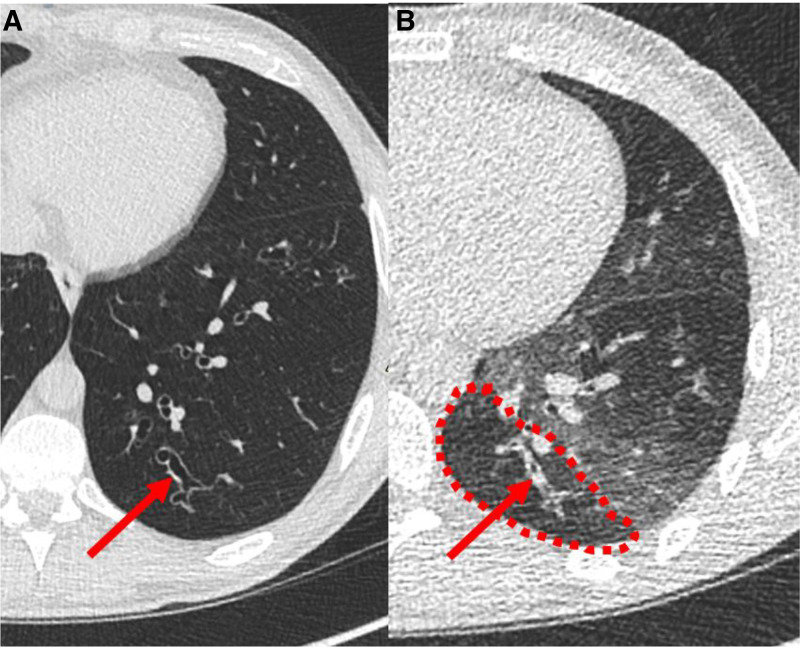
Axial computed tomography (CT) with inspiratory image left (*A*) and expiratory image right (*B*), subsegmental posterior-basal left lower lobe. The airway pointed out with the red arrow is patent, but ectatic on the inspiratory image, but collapses on the forced expiratory image. The adjacent low attenuation (inside red dashed line) is generally interpreted as “small airways disease” or “trapped air,” but the nonphysiologic larger airways collapse could contribute to this appearance. This participant had a percent predicted forced expiratory volume in 1 second (ppFEV_1_) of 83% and *Z*-score of −1.36. The location of the collapsed airway is at the 6th generation, which is still within the proximal airways.

Here, we use the trapped air % from spiro-CT as the “gold standard” to compare to pulmonary function parameters; however, spiro-CT gives only limited information about this pathological feature (22). When scoring trapped air % from spiro-CT there are varying degrees of the severity of trapped air, which can be observed when reviewing the Hounsfield units from these spaces (23). Density analysis can be performed using differing Hounsfield unit cuts offs, but there is no standardized method, particularly in pediatrics. As we found with the 5IC and 5VC methods, collateral ventilation and lung units with time constants slower than tidally ventilated lung units play a varying role for different individuals likely owing to variation in disease conditions and severity (24). Therefore, we consider the term “trapped air” a misnomer which should be updated to UVLU. The low attenuation (dark/“trapped air”) appearance that is scored on spiro-CT could also represent localized hypoperfusion secondary to local hypoxia rather than differences in ventilation (25). However, hypoperfusion could also help explain the mechanism by which collateral ventilation channels are activated. Hypoperfusion is an evolutionary mechanism by which increased concentrations of CO_2_ in a poorly ventilated/obstructed lung space reduces the resistance in collateral airways, and therefore increases collateral ventilation. For example, when 5% CO_2_ is infused into an obstructed area of lung, the resistance to collateral ventilation channels decreases significantly by ∼45% compared with when it is infused with medical air (26). We hypothesize that this is one of the mechanisms that may be at play here and helps explain why modeled N_2_ concentrations are so much lower than the assumed 78%. We also recognize that trapped air % is scored on lung slices after full expiration to residual volume. Forced expiratory maneuvers create very high and nonphysiologic intrathoracic pressures that could potentially result in the collapse of peripheral airways, particularly bronchioles that have no cartilage in the airway wall (Fig. 5). There is increasing attention on functional lung MRI to assess this pathophysiology (27), and such techniques could eventually become a new “gold standard.” There are number of different techniques and protocols, but the outcome most commonly used is ventilation defect percentage (VDP), which is akin to trapped air %. VDP is dependent on the gas used, for example, VDP was higher with ^129^Xe MRI than with ^3^He MRI when both techniques were used on the same patients on the same day; this was hypothesized to be related to particle size and diffusive properties of each gas (28). Although attention has mainly been on hyperpolarized gas MRI, we are studying the clinimetric properties of oxygen enhanced MRI, which rather than a single-breath hold, uses free breathing of 100% oxygen over a 4-min period. The free breathing protocol allows for a better understanding of ventilation in and around obstructed lung units and the process of collateral ventilation can be easily observed (29).

### Limitations

Our study is not without limitations: first, we studied only a small group of pwCF at a single time point. Participants were not naïve to MBW_ShX_ or the 5IC method, but none had previously performed the 5VC method. A limitation of the single breath slow vital capacity for MBW_ShX_ is that severe disease may be underestimated, and that more than one breath may be needed to accurately assess UVLU. We also recognize that without sampling the individual lung compartments directly there is no way to be sure of N_2_ concentrations. However, we have demonstrated the large variability between patients, an important finding to highlight. For three patients, we could not model an appropriate N_2_ concentration as they had a higher volume of trapped gas from either 5-breath method than the measured amount by spiro-CT.

### Future Work

Work is underway to directly compare all the parameters in this study with oxygen-enhanced MRI, to further assess the UVLU pathophysiology. We are developing a UVLU calculation for both 5-breath methods and assessing whether it yields a better relationship with oxygen enhanced-MRI and spiro-CT. We are also exploring more sensitive CT analysis to assess the varying degrees of “trapped air” using Hounsfield units to better understand the variations between UVLU spaces and patients.

### Conclusions

The parameters from MBW_ShX_ demonstrated significantly stronger correlations with spiro-CT and better sensitivity and accuracy than the other pulmonary function methods assessed in this study. We consider the term “trapped air” either in the use of imaging or pulmonary function testing, something of a misnomer which can lead to an inaccurate assessment of an important physiological feature. We therefore propose the term underventilated lung units to be used instead, and MBW_ShX_ shows promise to assess this important pathophysiological feature.

## DATA AVAILABILITY

Data will be made available upon reasonable request.

## SUPPLEMENTAL MATERIAL

10.6084/m9.figshare.26362468 Supplemental Material and Supplemental Fig. S3: https://doi.org/10.6084/m9.figshare.26362468.

## GRANTS

This study was supported by the National Institute of Health Research (NIHR) through a Senior Investigator Award (to J.C.D.), the Royal Brompton Clinical Research Facility and the Imperial Biomedical Research Centre. This study was also supported by Cystic Fibrosis Foundation under Grant MCNALL19K0, by Cystic Fibrosis Trust under Grant VIA083, and by Cystic Fibrosis Ireland under Grant RECOVER01.

## DISCLOSURES

Harm Tiddens reports grants from Vectura Group Plc., other from Roche and Novartis, grants from CFF, Vertex, Gilead and Chiesi, outside the submitted work. In addition, Harm Tiddens has a patent Vectura licensed, and a patent PRAGMA-CF scoring system issued and is heading the Erasmus MC-Sophia Children’s Hospital Core Laboratory Lung Analysis. Paul McNally reports independent grants and speaker/board honoraria from Vertex outside the submitted work. Jane C Davies and her institution have received fees for Advisory Board participation, clinical trial leadership, and speaking engagements from Vertex Pharmaceuticals in the field of CFTR modulators but not directly related to this study, and from AbbVie, Arcturus, Boehringer Ingelheim, Eloox, Enterprise Thearpeutics and Novartis outside the scope of this work. None of the other authors has any conflicts of interest, financial or otherwise, to disclose.

## AUTHOR CONTRIBUTIONS

C.S. conceived and designed research; C.S. performed experiments; C.S. analyzed data; C.S., T.S., M.A., S.P., M.R., H.T., A.B., and J.C.D. interpreted results of experiments; C.S. prepared figures; C.S. drafted manuscript; C.S., T.S., M.A., S.P., M.R., P.M., H.T., D.C., A.B., and J.C.D. edited and revised manuscript; C.S., T.S., M.A., S.P., M.R., P.M., H.T., D.C., A.B., and J.C.D. approved final version of manuscript.
